# Antiquity of Excisions

**Published:** 1872-11

**Authors:** 


*American Journal of Medical Sciences.*

---

### THE ANTIQUITY OF EXCISIONS.

*To the Editor of the Medical Record:*

SIR: In the *American Journal of Medical Sciences,* for October, 1872, there appears quite a clever review of Markoe's "Treatise on Diseases of the Bones," in which the reviewer, on pp. 453–4, corrects Dr. Markoe's historical allusion to excision, and says: "White's operation, such as it was, was preceded by Filkin's excision of the knee in 1762." From this statement it might be inferred that a knowledge of the operations of excision performed prior to 1762 is not generally extant. That excision was known and practised a long time before the above mentioned date the following facts will go to prove: Mr. Le Cat, Professor of Anatomy and Surgery, communicated an account of his operation to the academy at Rouen, in the year 1751. The case occurred in the person of Charles Lehee, a child of three years old, of the village of Pitre, etc., from whom was extracted an entire tibia, exostosed and carious in its whole extent, between the two articulations, which had remained sound: the great deficiency of bone-substance was entirely supplied again by nature, and the patient acquired a new tibia, much firmer than that which he had lost. The same operator relates an account of the extraction of three inches and ten lines of the bone of the upper arm, which was followed by a regeneration of the bony matter. This performance, Mr. Le Cat tells us, he determined upon after considering the case of Charles Lehee, which instance had sufficiently convinced him that bones have the power to regenerate. The subject was a soldier, Francis Romain, aged forty-one, who it appears received at the battle of Rocou, in 1755, a gunshot wound such as modern surgeons would consider to be a typical case for excision of the shoulder joint. The operation was done in 1760, and on October 12, Romain was discharged from hospital, very thankful for the recovery of his health and the perfect cure he received. A detailed account of these cases, with a description of a machine made use of to keep the upper and lower pieces of the bone at their proper distances, during the time that the regeneration was taking place, can be found in the *Philosophical Transactions*, vol. lvi., 1766, p. 270, *et seq.*

Tracing the history of excisions a little further back, we read:* Ansam inventionis hujus præbuisse videtur Thomas, chirurgus anglicus, qui Pezenate Languedociæ, anno 1740, puellæ quatuor annorum, humeri necrosi laborantis, curam in se susceperat; caput columque humeri, necrosi destructa, e vulnere, quod abcessus articuli aperiendi causa fecerat, prominebant, quæ reponere quidem studuit, quæ vero posthac necessitate coactus amovit; quod vix speravit factum; puella uno

* Ullrich (G. E.)—*De Ossium Resectione*, Lipsæ: 1857.

mense præterlapso sanata ita est, ut ætatis anno decimo quinto omnibus famulæ officiis fungi valeret.

Long before White's operation, Diemerbroeck (1637) mentions a case of fracture where a part of the tibia was removed by the saw, and hardness restored so far that the bone absolutely recovered its original firmness. But even he was not the first to perform this operation, for in his book he mentions a similar operation, which was done a little after the seige of Ostend was instituted in 1601. He writes: Molitor quidam e mola sua decidens, tibiam cum fibula in medio confregit, tanta cum violentia, ut superior pars carnem perforans, in ipsam duram terram vi adigereter, atque non solum carne, verum etiam periosteo privaretur; senex quidam chirurgus, in arte sua experimentissimus, qui olim obsidioni Ostendæ famosæ interfuerat, suasit istius ossis, periostoe circiter ad duorum dignitorum latitudinem penitus denudati, ablationem mox consentientibus cæteris chirurgus: dictum factum, et secunda religatione os illud subtili serra amputatum fuit: tunc dictus chirurgus crus ad pristinam longitudinem rursus extendit, et sic in ligneatheca æqualiter deposuit, vulnus quotidie sine cruris commotione inspiciendo: atque six ex utroque ossis fine paullatim succrescens callus ita coibat, ac sensim in osseam duritiem firmabatur, ita ut crus pristinam longitudinem retineret, et ubi os ablatum esset, postea videri vix posset.*

For the curious it may be interesting to go back still further. The following is a translation from a Polish work on Operative Surgery:† Excision was already known in ancient times, and we have undoubted proof of this in the writings of Hippocrates, Celsus, Avicena, Galen, and Paulus Æginitus. In Hippocrates' work, *De Articulis*, we find: *At resectiones ossium perfectæ circa articulos et in manu, et in tibia ad malleolos, et in cubitu ad juncturam manus, plerisquæ quibus resecantur innoxiæ sunt, si non statim animi deliguium evertat, aut quarto die, febris continua accedat.*

When, in after times, every science fell into decline, this operation became lost: and if, in subsequent centuries, excision was ever employed, the operators have never known how to de-

---

* Bercht (Alexander)—*De Resectione Articulorum.* Berolini: 1849.

† Le Brun, *Chirurgia Operacjna.* Warszawa: 1868, p. 442.

scribe it with accuracy. Duhamel's investigations (1739) into the destination of the periosteum in vivification and growth of the bone, which led him to the opinion that " the periosteum forms the bone," gave the first impulse in the revival of the operation, already justified by the physiological and anatomical knowledge of that time.

Before concluding, it may be remarked that the reviewer, in criticising " the peculiarities of Dr. Markoe's style," selects for that purpose a sentence which he misquotes. In the copy of Dr. M.'s work before me, at page 157, is found the word "*presents*," in the sentence referred to, and not "*pursues*." I. C. R.

1735 F street, Washington, November 11, 1872.

